# A multi-fractured well performance model for unconventional reservoirs

**DOI:** 10.1016/j.heliyon.2022.e10827

**Published:** 2022-10-01

**Authors:** Shaibu Mohammed, Yaw Akyampon Boakye-Ansah, Ebenezer Gyamfi-Yeboah, Kwamena Opoku Duartey, Warden Ivan Nyamekye

**Affiliations:** Department of Petroleum and Natural Gas Engineering, University of Energy and Natural Resources, Sunyani, Ghana

**Keywords:** Unconventional reservoirs, Duong's model, Extended Duong's model, Estimated ultimate recovery, Future performance prediction, Boundary-dominated flow

## Abstract

Due to the ultra-low permeability of unconventional reservoirs, transient state prevails for a considerable period. Despite this, fracture interference can cause an apparent no-flow boundary. Consequently, the Duong's model, which was developed for transient-state period, yields unreliable estimates during the late-time period. In this paper, the Duong's model is modified to account for boundary effects caused by fracture interference and/or unstimulated reservoir regions that serve as no-flow boundaries. Specifically, an empirical correction function, which assumes an exponential decline, has been used as a “modifier” to extend the Duong's model to boundary-dominated flow period. The correction function ensures that during boundary-dominated flow period, an exponential-decline behaviour dominates. The proposed rate-decline model encompasses a gamma function, which converges at large times. Results show that a fractured-well production behaviour is characterised by a decaying power-law during early-time period and tends to exponential decline during late-time period. The results also suggest that although the conventional Duong's model gives good estimates during the transient-state period, it yields optimistic estimates during the boundary-dominated flow period. The proposed model gives a good match and estimates not only in the transient-state period, but also in the boundary-dominated flow period. A major advantage of the proposed model is that it converges to estimated ultimate recovery at large times without imposing any rate and time limits. A good agreement of the estimated ultimate recovery with analytical and semi-analytical models was obtained. Also, results suggest that the proposed model gives conservative estimates. The proposed model will be useful for analysing and predicting both the early- and late-time production performance of a multi-fractured well producing from an unconventional reservoir.

## Highlights

• An empirical model that extends the Duong's model to late-time period is formulated.
• A power-law decline is a characteristic of early-time period.
• An exponential decline is a characteristic of late-time period.
• The proposed model converges to the ultimate recovery at large times.

## Nomenclature

a: Duong's model parameter day^−1^
b: Extended Duong's model parameter dimensionless
c: Extended Duong's model parameter dimensionless
*EUR*: Estimated ultimate recovery STB (Bstb) or Mscf (or Bscf)
fc: Correction function dimensionless
m: Duong's model parameter dimensionless
n: Conventional model transient-decline exponent dimensionless
Q: Cumulative production STB or Mscf
Q1: Cumulative production at day 1 STB or Mscf
q: Production rate STB/D or Mscf/D
$q_{\mathrm{eco}}$: Economic rate STB/D or Mscf/D
q1: Production rate at day 1 STB/D or Mscf/D
**RF**: Recovery factor Dimensionless
t: Time Day, or month or year
$t_{\mathrm{eco}}$: Economic or abandonment time days
t(a,b,c): Time function (based on Eq. (11b)) dimensionless
τ: Characteristic time day, month, or year

## 1 Introduction

Unconventional reservoirs have sustained hydrocarbon production to meet the world's energy demand. This trend may continue in the (un)foreseeable future. However, unconventional reservoirs pose many challenges. For instance, they are characterised by extremely low permeability, and so require massive hydraulic fracturing and horizontal well technology. Further, these unconventional reservoirs are stress-sensitive, and so require complex geomechanical modelling. Moreover, the storage and transport capacity of unconventional reservoirs are complex.

The gas-in-place of shale formation includes free, adsorbed, and absorbed gas (Aguilera, 2016 and Ambrose et al., 2012). The free gas is stored in the pore volume of the formation; the adsorbed gas is adhered to the surfaces of the pore walls; and the absorbed gas is dissolved in hydrocarbon liquid and formation water (Ambrose et al., 2012). According to Aguilera (2014), free gas can also be stored in hydraulic fractures. Other findings have suggested that free gas is stored in the pores of organic matter (Ruppel and Loucks, 2008, Wang and Reed, 2009). While Ambrose et al. (2012) argues that free gas in shale formation is overestimated, Aguilera (2016) contends that significant amount of free gas that are stored in organic matter, natural and induced fractures, are not taken into consideration when estimating shale gas-in-place.

Knudsen diffusion, surface diffusion and molecular diffusion play crucial roles regarding the transport mechanism of unconventional reservoirs (Javadpour et al., 2007, Javadpour, 2009) while Darcy's equation has been used to model fluid flow in hydraulic fractures (Brown et al., 2009, Ozkan et al., 2009). Thus, gas transport in unconventional reservoirs occurs at different scales.

Due to these complex storage and transport mechanisms, models for conventional reservoirs are inadequate for unconventional reservoirs. Therefore, several attempts have been made to develop models and analysis methods for unconventional reservoirs. These include analytical solutions (Wattenbarger et al., 1998, Male, 2019), semi-analytical solutions (El-Banbi and Wattenbarger, 1998, Bump and McKee, 1988, Brown et al., 2009, Ozkan et al., 2009, Patzek et al., 2013, Ahmadi et al., 2021), numerical solutions, and empirical solutions (Ilk et al., 2008, Valko, 2009, Valko and Lee, 2010, Clark et al., 2011; Duong, 2011 and Mohammed et al., 2021). Although the effects of rock and fluid properties are determined by analytical solutions, these solutions make several unrealistic assumptions that render them inadequate, especially for unconventional reservoirs. Furthermore, these models require several input parameters, most of which are not readily available. In consequence, although analytical, numerical, and semi-analytical models provide useful information about the impact and sensitivity of rock and fracture parameters, the unavailability and uncertainties of these parameters limit the applications of these models.

Empirical models have been used to complement analytical and numerical solutions. Even though empirical models do not consider the physics of fluid flow in porous media, and thus the model parameters are not explicitly related to reservoir and fracture parameters, they have many other advantages (Valko, 2009, Valko and Lee, 2010, Duong, 2011). For example, empirical models require few input parameters, which can be estimated with production and pressure data; also, they can be used for estimating ultimate recovery; furthermore, they are useful for production forecasting even without reservoir and fracture parameters; in addition, they have been used for statistical analysis (Valko, 2009, Valko and Lee, 2010, Duong, 2011). Due to these advantages, empirical models have been developed for unconventional reservoirs. Ilk et al. (2008) presented a power-law exponential model to analyse and predict unconventional reservoir performance. The authors found that during early-time period, the production behaviour of a fractured well assumes a power law, and then transitions to exponential decay during the boundary-dominated flow (BDF). Valko (2009) proposed that unconventional reservoir performance could be analysed with a stretched exponential model. Clark et al. (2011) presented a logistic growth model for unconventional reservoir. The model puts a threshold on the cumulative production. Duong (2011) proposed a rate-decline model for a fractured well in unconventional reservoirs.

Due to the extremely low permeability of unconventional reservoirs, transient state prevails for a considerable period (Wattenbarger et al., 1998 and Taji and Alp, 2021); in this instance, the Duong's model may be used. However, due to fracture interference, an apparent no-flow boundary prevails (Joshi and Lee, 2013 and Kanfar and Wattenbarger, 2012); in this instance, the Duong's model over-predicts a well future performance, and so it becomes inapplicable (Kanfar and Wattenbarger, 2012, Mohammed et al., 2021). Consequently, attempts have been made to extend the Duong's model to boundary-dominated flow period (Joshi and Lee, 2013; Cauter, 2013 and Mohammed et al., 2021). Unfortunately, these methods require one to switch from the Duong's model to the Arps' hyperbolic model when BDF is reached, as well as impose a limit on the reserve at late times. The decline rate at which such switch is required is chosen arbitrarily.

In this paper, we extend the Duong's model to BDF period. The aim is to analyse and predict not only the transient-state period, but also the BDF period. This is worth considering because, even though unconventional reservoirs exhibit a prolonged transient state-period, the effect of a no-flow boundary is felt eventually due to fracture interference (Kanfar and Wattenbarger, 2012 and Mohammed et al., 2021).

## 2 Background

During transient-state period, a fractured-well performance is characterised by a decaying power law. The governing equation for such period given by (Wattenbarger et al., 1998):

(1) $$q=q_{1}t^{-n}$$

Eq. (1) suggests that a log-log plot of *q* vs *t* will yield a straight-line with a negative slope, −*n*, and an intercept, $q_{1}$. Eq. (1) is strictly used for early-time period; in consequence, it gives unreliable estimates at late-time period (Kanfar and Wattenbarger, 2012). Also, Eq. (1) was derived based on conventional reservoirs; therefore, it is unsuitable for unconventional reservoirs (Duong, 2011, Kanfar and Wattenbarger, 2012 and Male, 2019). Consequently, Duong (2011) proposed a rate-decline model exclusively for a fracture-dominated flow in unconventional reservoirs which is given as:

(2) $$\frac{q}{Q}=at^{-m}$$

**Figure 1:**
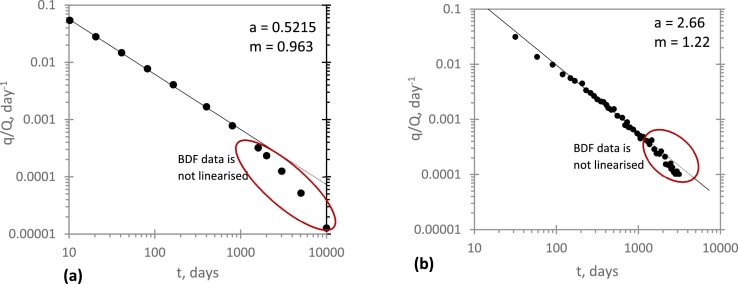
Application of Duong's model to: (*a*) simulated data; *b* field data.

In Fig. 1, an application of Duong's model to production performance of a simulated well (Fig. 1a) and field data from a real well (Fig. 1b) is shown. Although Duong's model linearises the transient-state data, the BDF data is not linearised. This suggests that Duong's model yields optimistic estimates during BDF period. Though not shown here, we have made similar observations with a number of field data. These results corroborate with previous findings (Kanfar and Wattenbarger, 2012, Meyet et al., 2013 and Mohammed et al., 2021).

## 3 Extended Duong's model

Due to the drawback of Duong's model earlier mentioned, here we propose an alternated model called extended Duong's model to account for boundary effect which is given as:

(3a) $$\frac{q}{Q}=at^{-m}f_{c}$$

where the correction function, $f_{c}$ which assumes an exponential decay is given as:

(3b) $$f_{c}=\exp(-ct)$$

In Eq. (3a) and (3b), *a* and *m* are transient-decline parameters, which govern depletion path during transient-state period; *c* is the BDF-decline exponent, which governs depletion path during BDF period. *c* is so small (c≪1) that at early time $f_{c}\approx 1$; therefore, at early time, the extended Duong's model (Eq. (3a)) and the Duong's model (Eq. (2)) become identical. At late time, however, the exponential function dominates.

**Figure 2:**
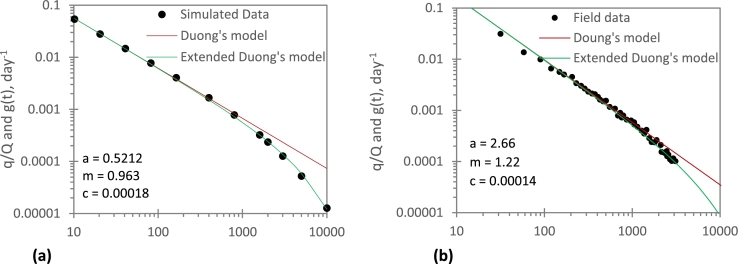
Application of extended Duong's model to: (*a*) simulated data; (*b*) field data.

To validate Eq. (3a) (i.e., the extended Duong's model (EDM)), simulated and field data in Fig. 1 was used. In Fig. 2, an application of the EDM is demonstrated. The EDM matches not only the transient-state data, but also the BDF data. Thus, the correction function (Eq. (3b)) extends the conventional Duong's model so that not only the transient-state data can be analysed, but also the BDF data.

On the one hand, the Duong's plot (i.e., a log-log plot of $at^{-m}$ vs *t*) linearises the transient-state data but it is unable to match the BDF data (Fig. 2). On the other hand, the extended Duong's plot [a log-log plot of $g\left(t\right)(=at^{-m}f_{c})$ vs *t*] linearises the transient-state data and tends to exponential decline during the BDF period (Fig. 2). This finding suggests that the correction function provides a simple yet a useful technique to account for the BDF regime. The procedure for estimating *c*, and hence predicting the future reservoir performance, will be presented later in this paper.

### 3.1 Rate-time and cumulative-time relations

Here, we formulate rate-time and cumulative-time relations of the extended Duong's model. These relations are used for the analysis and prediction of early- and late-time future production performance of a multi-fractured well.

The relation between production rate and cumulative production is given as:

(4) $$q=\frac{dQ}{dt}$$

Substitution of Eq. (4) into Eq. (3a) gives:

(5) $$\frac{dQ}{dt}=aQt^{-m}e^{-ct}$$

Letting m=1−b and using the initial condition (i.e., $Q(t=1)=Q_{1}$), the integral of Eq. (5) gives:

(6) $$\ln\left(\frac{Q}{Q_{1}}\right)=a\int_{1}^{t}t^{b-1}e^{-ct}dt^{\prime }$$

Eq. (6) can also be expressed as:

(7) $$\ln\left(\frac{Q}{Q_{1}}\right)=a\left(\int_{0}^{t}t^{b-1}e^{-ct}dt^{\prime }-\int_{0}^{1}t^{b-1}e^{-ct}dt^{\prime }\right)$$

Solving for the cumulative production, Eq. (7) gives:

(8) $$Q=Q_{1}\exp\left\{\frac{a}{c^{b}}\left[\gamma \left(b,ct\right)-\gamma \left(b,c\right)\right]\right\}$$

Now, differentiating Eq. (8) with respect to time gives:

(9) $$\frac{dQ}{dt}=Q_{1}at^{-(1-b)}\exp\left\{\frac{a}{c^{b}}\left[\gamma \left(b,ct\right)-\gamma \left(b,c\right)\right]-ct\right\}$$

Then, from Eq. (9), the production rate model is:

(10) $$q=q_{1}t^{-(1-b)}\exp\left\{\frac{a}{c^{b}}\left[\gamma \left(b,ct\right)-\gamma \left(b,c\right)\right]-ct\right\}$$

Equations (8) and (10) constitute the cumulative-time and rate-time relations of the extended Duong's model, respectively. In these relations, *a* and *b* are the model parameters that govern transient-state period; *c* is the model parameter that governs boundary-dominated flow period; $q_{1}(=Q_{1}a)$ is the production rate at t = 1 day (month or year); γ(b,ct) and γ(b,c) are the lower incomplete gamma function.

It should be noted that the proposed model (Eq. (10)) is based on a unit solution. In the Appendix, we have demonstrated that provided the unit solution is adhered to, the cumulative-time and rate-time relations are dimensionally consistent; this is also true for the case of the conventional Duong's model.

The extended Duong's model assumes a single–phase flow (either oil or gas), constant-pressure production, vertical/horizontal fractured well in unconventional reservoirs. The data that we have analysed include low-permeability sandstones and shale formations.

For unconventional reservoirs, 1<m<2; hence, *b* is a negative non-integer number; specifically, −1<b<0. Therefore, by virtue of the recurrence relation, the lower incomplete gamma function can be evaluated. For conventional reservoirs, 0<m<1; thus, in the case of conventional reservoirs, *b* is a positive non-integer number. Therefore, the proposed model can also be applied to conventional reservoirs.

In Table 1, we compare the formulations of the Duong's model and the extended Duong's model. The extended Duong's model encompasses a gamma function, which offers an advantage over the Duong's model regarding convergence to estimated ultimate recovery (EUR).

**Table 1:** Duong's and the extended Duong's formulations.

Variable	Duong's model	Extended Duong's model
*q*/*Q*	*at*^−(1−*b*)^	$at^{-(1-b)}\exp(-ct)$
*q*	$q_{1}t^{-(1-b)}\exp\left[\frac{a}{b}(t^{b}-1)\right]$	$q_{1}t^{-(1-b)}\exp\left\{\frac{a}{c^{b}}\left[\gamma \left(b,ct\right)-\gamma \left(b,c\right)\right]-ct\right\}$
*Q*	$\left(q_{1}/a\right)\;\exp\left[\frac{a}{b}(t^{b}-1)\right]$	$\left(q_{1}/a\right)\;\exp\left\{\frac{a}{c^{b}}\left[\gamma \left(b,ct\right)-\gamma \left(b,c\right)\right]\right\}$
EUR	$\left(q_{eco}/a\right)t_{eco}^{1-b}$	$\left(q_{1}/a\right)\;\exp\left\{\frac{a}{c^{b}}\left[\Gamma \left(b\right)-\gamma \left(b,c\right)\right]\right\}$
RF = *Q*/EUR	$\left(q/q_{eco}\right){\left(t/t_{eco}\right)}^{1-b}$	$\exp\left\{-\frac{a}{c^{b}}\left[\Gamma \left(b\right)-\gamma \left(b,ct\right)\right]\right\}$

### 3.2 Analysis procedure

Here, we present the analysis procedure of the extended Duong's model. The analysis method is used for model parameters estimation and production prediction.

1. ***Estimation of a and b***: Based on Duong's model, plot q/Q against *t* on a log-log graph. This plot linearises transient-state data; hence, such plot is used to estimate *a* and *b*.
2. ***Estimation of c***: As an initial guess, assume a value for *c*. Then, based on Eq. (3a), compute $g\left(t\right)\left(=at^{-(1-b)}e^{-ct}\right)$. Next, use nonlinear regression to determine *c*. In this study, Microsoft Excel nonlinear Solver was used. Note that while *a* and *b* are estimated with transient-state data, *c* is estimated with BDF data.
3. ***Estimation of***$q_{1}$: Based on Eq. (10), construct a linear plot of *q* against t(a,b,c). Eq. (10) can be re-written as:

(11a) $$q=q_{1}t\left(a,b,c\right),$$

where,

(11b) $$t\left(a,b,c\right)=t^{-(1-b)}\exp\left\{\frac{a}{c^{b}}\left[\gamma \left(b,ct\right)-\gamma \left(b,c\right)\right]-ct\right\}$$

A linear plot of *q* vs t(a,b,c) yields a straight-line through the origin during the transient-state period; thus, $q_{1}$ is estimated with transient-state data.

4. ***Estimation of EUR***: At large time (i.e., as t→∞), the estimated ultimate recovery (EUR) is determined with the following equation (deduced from Eq. (8)):

(12) $$\mathrm{EUR}=\frac{q_{1}}{a}\exp\left\{\frac{a}{c^{b}}\left[\Gamma \left(b\right)-\gamma \left(b,c\right)\right]\right\}$$

5. ***Production Prediction***: Eq. (11a) can then be used to predict the future production performance; Eq. (13) is used for predicting the recovery factor (RF):

(13) $$\mathrm{RF}(t)=\frac{Q}{\mathrm{EUR}}=\exp\left\{\frac{a}{c^{b}}\left[\gamma \left(b,ct\right)-\Gamma \left(b\right)\right]\right\}$$

Eq. (13) has been formulated by taking the ratio of Eqs. (8) and (12).

## 4 Results and discussion

In this section, the extended Duong's model is validated with a numerical data and applied to field data. The aim is to assert our argument that the proposed model does extend the conventional Duong's model to the BDF period. Also, the bounded nature of the model at large times is discussed.

### 4.1 Model validation

Here, we validate the EDM using a numerical data of a fractured well. The numerical data was taken from Carter (1985, Table 1). The initial gas-in-place for this case is 3.087 Bscf.

**Figure 3:**
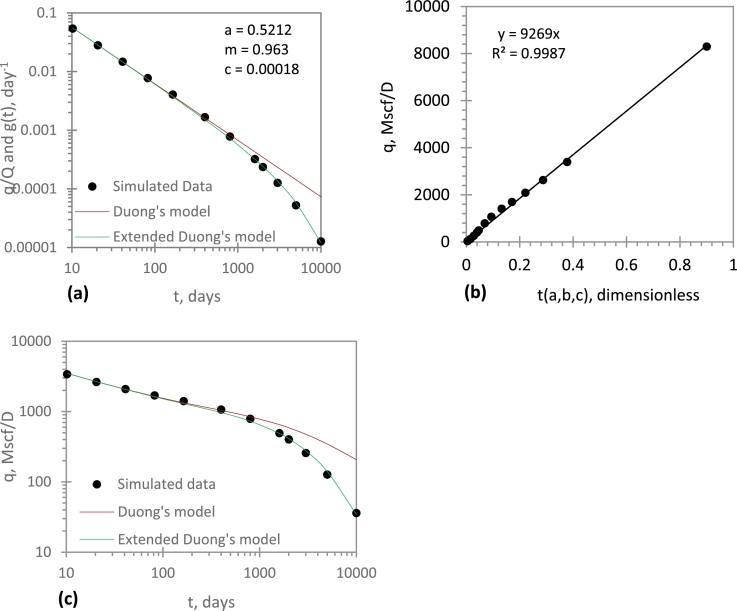
Performance analysis of EDM for a simulated data: (*a*) a, m, c estimation; (*b*) *q*_1_ estimation; (*c*) History matching and performance prediction.

Fig. 3a is a log-log plot of q/Q against time based on Duong's model. Such plot linearises the transient-state data; therefore, the parameters *a* and *m* (and hence *b*) are determined from the intercept and slope, respectively. Then, a nonlinear regression method was used to determine *c*. The *c* value that gave a good match was *0.00018*.

Next, a linear plot of *q* vs t(a,b,c) is constructed (Fig. 3b), and $q_{1}$ is determined from the slope; the transient-state data is used to determine $q_{1}$.

Having estimated the model parameters (a,b,c, and $q_{1}$), the future production performance can be forecasted with Eq. (11a). This is shown in Fig. 3c; the Duong's model has been included for the sake of comparison. Although the Duong's model gives a good match during the transient-state period, it overestimates the production during BDF period. The EDM gives a good match during the transient state and BDF period. Based on Eq. (12), the EUR is 2.736 Bscf; this occurs at 10,000 days (27 years). Unfortunately, the Duong's model cannot be used to compute the EUR until rate and time limits are imposed.

### 4.2 Application

In this section, we apply the extended Duong's model to field data for three different cases.

#### 4.2.1 Case 1

**Figure 4:**
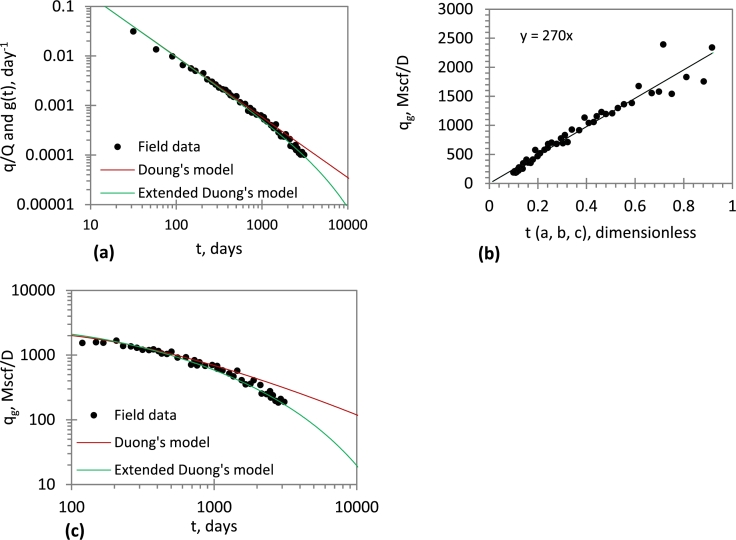
Application of EDM to field data: (*a*) a, m, c estimation; (*b*) *q*_1_ estimation; (*c*) History matching and performance prediction.

The production data (West Virginia, Well B) was previously analysed by Fraim and Wattenbarger (1987). The performance of the Duong's model and extended Duong's model is compared (as shown in Fig. 4a and c). Fig. 4b shows a linear plot of *q* vs t(a,b,c) is constructed; $q_{1}$ is determined from the slope. The extended Duong's model (EDM) gives a good match at both early and late times. In contrast, Duong's model gives a poor match during the late time, although it produces a good match during the early time. This finding suggests that, when boundary-dominated flow has been reached, Duong's model becomes inapplicable. However, the proposed model is applicable during the transient-state period, as well as the boundary-dominated flow period.

In Table 2, we compare the results obtained for the estimated ultimate recovery with some conventional models. Mohammed and Enty (2013) used a flowing material balance; Ansah et al. (2000) used a semi-analytical model; Blasingame and Lee (1988) used an analytical model; and Fraim and Wattenbarger (1987) used type curve analysis. The agreement of the estimate of EDM with conventional models validates EDM even though EDM circumvents the use of pseudo time, which relies on a time-consuming iteration technique.

**Table 2:** Comparison of model results for *Case 1*.

Authors	Fetkovich et al. (1987)	Fraim and Wattenbarger (1987)	Blasingame and Lee (1988)	Ansah et al. (2000)	Mohammed and Enty (2013)	EDM
						(This work)
EUR (Bscf)	3.36	3.3	2.63	2.85	2.78	2.35

#### 4.2.2 Case 2

The data (Well 5 lease number 146045) in this case was obtained from Railroad Commission of Texas (2022). It spans from July, 1993 to January, 2021.

**Figure 5:**
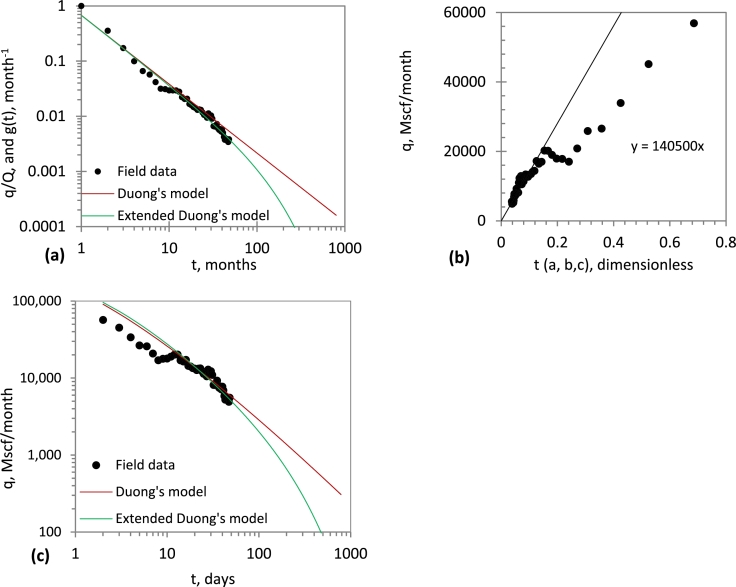
Application of EDM to field data: (*a*) a, m, c estimation; (*b*) *q*_1_ estimation; (*c*) History matching and performance prediction.

The Duong's model and the extended Duong's model are compared (Fig. 5a and c). A linear plot of *q* vs t(a,b,c) is constructed (Fig. 5b), and $q_{1}$ is determined from the slope. Both models give good match during the transient-state period; the extended Duong's model tends to an exponential decline, and thus gives a good match during the boundary-dominated flow period. The early-time data that deviate may be due to production from a previous fracture treatment, or may be due to skin.

The results in Fig. 5 affirms our argument that the extended Duong's model improves the conventional Duong's model; it does so by extending the Duong's model to BDF period.

#### 4.2.3 Case 3

**Figure 6:**
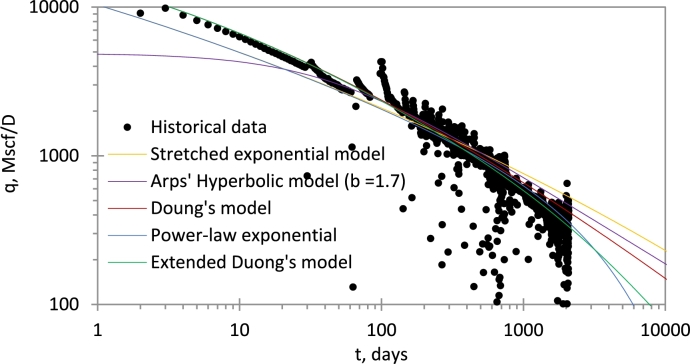
Performance history and forecasting of EDM and conventional models.

The data in Case 3 was obtained from Blasingame (2022). In Fig. 6, we compare the performance of EDM and conventional models. The power-law exponential (PLE) model is the most conservative, followed by the extended Duong's model. The extended Duong's model, however, gives the best match. The Duong's model overestimates production rate at late time.

Cumulative production for the various models for rate and time limits of 30 years and 10 Mscf/D, respectively, is presented in Table 3. EDM and PLE are the most conservative. The advantage that EDM has over PLE is the explicit nature of the cumulative production of EDM. Also, notice that the extended Duong's model outperforms the Duong's model, as well as the stretched exponential and hyperbolic models, at late time.

**Table 3:** Comparison of reserve estimates among models for *Case 3*.

Time limit	30	Years
Rate limit	10	Mscf/D
	Gp, Bscf	
Model	Time limit	Rate limit
Stretched exponential	2.57	3.76
Hyperbolic (b = 1.7)	2.44	3.35
Duong's model	2.37	3.04
Extended Duong's model	2.16	2.78
Power-law exponential	2.08	2.72

**Conclusion**

The purpose of this paper was to extend the Duong's model to boundary-dominated flow period. The following conclusions are deduced from this study:

1. The production behaviour of a multi-fractured well in an unconventional reservoir is characterized by a decaying power law at early times, and an exponential decline at late times;
2. The Duong's model gives good estimates during the early time; unfortunately, it gives optimistic results during late times;
3. A new rate-decline model that extends the Duong's model to late time has been proposed; the model relates the production rate to a gamma function, which bounds the cumulative production at late time;
4. A comparison with conventional models revealed that the proposed model gives conservative estimates;
5. Application of the proposed model to field data suggests that the model is suitable for the analysis and prediction of a fractured well in unconventional reservoirs.

## Declarations

### Author contribution statement

Shaibu Mohammed: Conceived and designed the experiments; Performed the experiments; Analyzed and interpreted the data; Wrote the paper.

Yaw Akyampon Boakye-Ansah: Conceived and designed the experiments; Analyzed and interpreted the data; Contributed reagents, materials, analysis tools or data; Wrote the paper.

Ebenezer Gyamfi-Yeboah: Performed the experiments; Analyzed and interpreted the data; Wrote the paper.

Kwamena Opoku Duartey: Performed the experiments; Analyzed and interpreted the data; Contributed reagents, materials, analysis tools or data.

Warden Ivan Nyamekye: Analyzed and interpreted the data; Contributed reagents, materials, analysis tools or data; Wrote the paper.

### Declaration of interests statement

The authors declare no conflict of interest.

### Data availability statement

Data included in article/supplementary material/referenced in article.

### Funding statement

This research did not receive any specific grant from funding agencies in the public, commercial, or not-for-profit sectors.

### Additional information

No additional information is available for this paper.

## Appendix A Units and dimensions of the extended Duong's model

Here, we shed light on the units and dimensions of the parameters of the extended Duong's model. This is important due to at least three reasons. Firstly, a dimensionally consistent model attests to the accuracy of the model. Secondly, we define the model parameters in terms of rock, fluid, and fracture parameters. Lastly, we show that general solutions, such as type curves, can be developed for the analysis and future performance prediction.

The extended Duong's model (Eq. (3a) in the main text) is:

(A.1) $$\frac{q}{Q}=at^{-m}\exp\left(-ct\right)$$

The left-hand side of Eq. (A.1) has a unit of day^−1^ (month^−1^ or year^−1^). Then, due to equality, the right-hand side should have the same unit. To ensure dimensional consistency, we introduce a characteristic time, and Eq. (A.1) therefore becomes:

(A.2) $$\frac{q}{Q}=a{\left(\frac{t}{\tau }\right)}^{-m}\exp\left(-c\frac{t}{\tau }\right)$$

In Eq. (A.2), *τ* is the characteristic time and has a dimension of time with unit as day (month or year); therefore, *a* has a dimension of a reciprocal of time and a unit of day^−1^; and *c* and *m* have no units and dimensions. With these units and dimensions, Eq. (A.2) is consistent in terms of units and dimensions.

Based on unit solution (τ=1), Eqs (A.2) reduces to Eq. (A.1); thus, considering the unit solution, the units and dimensions of the variables and parameters in Eqs (A.1) and (A.2) are identical. Consequently, the extended Duong's model (and of course, the original Duong's model) is based on a unit solution.
